# Suppression of AMP‐activated protein kinase reverses osteoprotegerin‐induced inhibition of osteoclast differentiation by reducing autophagy

**DOI:** 10.1111/cpr.12714

**Published:** 2019-11-07

**Authors:** Xishuai Tong, Chuang Zhang, Dong Wang, Ruilong Song, Yonggang Ma, Ying Cao, Hongyan Zhao, Jianchun Bian, Jianhong Gu, Zongping Liu

**Affiliations:** ^1^ College of Veterinary Medicine Yangzhou University Yangzhou Jiangsu China; ^2^ Jiangsu Co‐innovation Center for Prevention and Control of Important Animal Infectious Diseases and Zoonoses Yangzhou Jiangsu China; ^3^ Jiangsu Key Laboratory of Zoonosis Yangzhou Jiangsu China; ^4^ Joint International Research Laboratory of Agriculture and Agri‐Product Safety The Ministry of Education of China Yangzhou University Yangzhou Jiangsu China; ^5^ Key Laboratory of Neurodegeneration of Jiangsu and Ministry of Education Co‐innovation Center of Neurodegeneration Nantong University Nantong Jiangsu China

**Keywords:** AMP‐activated protein kinase (AMPK), mammalian target of rapamycin (mTOR), osteoclasts, osteoprotegerin, ribosomal protein S6 kinase beta‐1 (p70S6K)

## Abstract

**Objectives:**

Osteoclasts (OC) are unique terminally differentiated cells whose primary function is bone resorption. We previously showed that osteoprotegerin (OPG) inhibits OC differentiation in vitro by enhancing autophagy via the adenosine monophosphate‐activated protein kinase (AMPK)/mTOR/p70S6K signalling pathway in vitro. Here, we aimed to elucidate the mechanism of AMPK mediated autophagy to regulate OPG‐mediated inhibition of OC differentiation and identify potential therapeutic targets associated with bone loss.

**Materials and Methods:**

We used the AMPK activator AICAR to determine the relationship between AMPK activation and OC differentiation, and studied the role of AMPK‐mediated autophagy in OPG‐mediated inhibition of OC differentiation by using autophagy inhibitors or AMPK knockdown.

**Results:**

AMP‐activated protein kinase activation caused LC3II accumulation and weakened OC differentiation activity. In contrast, inactivation of autophagy by 3‐methyladenine or Bafilomycin A1 could attenuate OPG‐mediated inhibition of OC differentiation via the AMPK/mTOR/p70S6K signalling pathway. Furthermore, the AMPK inhibitor compound C and knockdown of AMPK impaired OPG‐mediated inhibition of OC differentiation by inducing autophagy.

**Conclusions:**

These results demonstrated that the AMPK signalling pathway functions as a critical regulator in the OPG‐mediated inhibition of OC differentiation, by inducing autophagy. Our results provide a basis for future bone‐related studies on the AMPK signalling pathway.

**Highlights**

1. Activation of AMPK by AICAR can inhibit OC differentiation.
2. Autophagy plays a key role in OPG inhibiting OC differentiation via AMPK/mTOR/p70S6K signalling pathway.
3. Suppression of autophagy by 3‐MA or BAF can attenuate OPG‐mediated inhibition of OC differentiation.
4. Suppression of AMPK reverses OPG‐mediated inhibition of OC differentiation.

## 1 INTRODUCTION

Osteoclasts (OCs) are multinucleated, terminally differentiated bone‐resorbing cells.^1^ Osteoprotegerin (OPG)/receptor activator of nuclear factor κB (RANK)/receptor activator of nuclear factor κB ligand (RANKL) system is an important "axis centre" and is involved in the regulation of OC formation. RANK/RANKL signalling pathway regulates OC differentiation in normal bone development and remodelling to improve bone turnover.^2^, ^3^ OPG is a secretory glycoprotein, which competes with RANKL to block RANKL‐RANK binding, thereby inhibiting OC differentiation.^4^ Macrophage colony‐stimulating factor (M‐CSF) is also involved in OC formation, which is a cytokine with important biological function.^5^ RANKL and M‐CSF mediate OC differentiation via downstream signalling pathways that regulate transcription factor c‐Fos, nuclear factor of activated T‐cell cytoplasmic 1 (NFATc1), and hydrolysed enzyme cathepsin K (CTSK). OPG indirectly inhibits OC differentiation and bone resorption activity, and could be used to regulate bone density and improve bone mass.^6^

Reactive oxygen species (ROS) are involved in many physiological intracellular redox states, which consist of radical or non‐radical oxygen species. The level of intracellular ROS was increased in RANKL‐stimulated OC differentiation from OC precursors by a series of signalling cascade involving tumour necrosis factor (TNF) receptor‐associated factor‐6 (TRAF‐6), Rac1 and nicotinamide adenine dinucleotide phosphate (NAPDH) oxidase 1 (Nox1).^7^, ^8^ Overproduction of ROS linked to many metabolic diseases including osteoporosis, which results in oxidative stress that is a not balance between OC and osteoblast (OB).^9^, ^10^, ^11^ Actually, oxidative stress has a highly regulatory role in bone remodelling process, which promotes OC resorption and bone loss by increasing the ratio of RANKL/OPG leads to suppression of osteoblastic mineralization and activation.^10^ *N*‐acetyl‐cysteine (NAC) is a cysteine analogue drug and an antioxidant, which inhibits oxidative stress against OC formation by reduction of ROS and inactivation of NF‐κB and TNF‐α expression.^10^, ^12^ Adenosine monophosphate‐activated protein kinase (AMPK) also play a critical role and is associated with oxidative stress, inflammation and tumorigenesis.^13^

Autophagy is regulated by autophagy‐related (Atg) genes in eukaryotic cells.^14^ Beclin1 is involved in autophagy,^15^ as well as OC formation and bone resorption via autophagy.^16^ Autophagy inhibition by 3‐methyladenine (3‐MA), LY294002 or Beclin1/Atg7 knockdown downregulated the OC marker gene (tartrate‐resistant acid phosphatase (TRAP) and CTSK).^17^ Microtubule‐associated light chain protein 3 (LC3), an autophagy marker protein, is conjugated to a highly lipophilic phosphatidylethanolamine (PE) moiety by other Atg (eg Atg5, Atg7 and Atg12) complexes to promote autophagosome formation.^16^, ^18^ Atg5 and Atg7 knockdown inhibited the expression of OC markers TRAP and CTSK during OC differentiation,^19^ and LC3 knockdown did not affect TRAP‐positive multinucleated cell formation, but suppressed actin ring formation, CTSK release and OC bone‐resorbing capacity.^16^ p62 knockout mice show complex signs caused by OC inactivation in vivo*,* 20 and p62 knockdown attenuated RANKL‐induced OC marker gene (NFATc1 and CTSK) expression in vitro.^21^

Mammalian target of rapamycin (mTOR) is a highly conserved serine/threonine protein kinase, which is part of the mTORC1 complex, along with regulatory‐associated protein of mTOR (Raptor), mammalian lethal with SEC13 protein 8 (mLST8 or GβL).^22^, ^23^ Rapamycin (a specific mTOR inhibitor) decreased the number of TRAP‐positive multinucleated cells^24^ and expression of CTSK and matrix metalloprotein‐9 (MMP‐9), RANK and NFATc1 in vitro*.* 25 Autophagy is a catabolic process and involves phagophore formation and subsequent fusion of the autophagosome with lysosomes.^26^ In OCs, the lysosomes are highly acidic (pH ~ 4.5) and degrade extracellular and intracellular material. The lysosome inhibitors Bafilomycin A1 (BAF; a specific vacuolar H^+^‐ATPase [V‐ATPase] inhibitor) stop the lysosomes fusing with autophagosomes to form autolysosomes, inhibiting bone resorption in OCs.^27^, ^28^, ^29^ BAF attenuates OC differentiation by increasing p62 levels, reducing intracellular acidification of OC.^30^

AMP‐activated protein kinase is controlled by the AMP: ATP ratio and its upstream signalling.^31^ AMPK is a critical regulator of bone homeostasis; genetic deletion of catalytic α‐subunits of AMPK in mice reduces bone mass by increasing bone formation and resorption. Further, when bone marrow‐derived macrophages (BMMs) from AMPKα deficient mice were used as OC progenitors (OCP) to induce osteoclastogenesis in vitro, deletion of AMPKα1 increased osteoclastogenesis‐associated gene expression and augmented RANK signalling, negatively regulating osteoclastogenesis.^32^ Inhibition of AMPK with compound C (Com C; dorsomorphin) and knockdown by AMPKα1 siRNA increased the number of TRAP‐positive multinucleated cells and bone resorption activity by RANKL induced via activation c‐Fos and NFATc1 in vitro*.* 33 mTOR is centrally regulated by upstream AMPK signalling pathways.^34^ mTOR phosphorylates its downstream target protein p70S6K to regulate cell proliferation and protein synthesis.^35^

Osteoprotegerin inhibited RANK mRNA expression in duck embryo bone marrow cells or OC‐like cells (OCLs).^36^ We previously demonstrated that OPG inhibits OC differentiation by enhancing autophagy via AMPK/mTOR/p70S6K in vitro.^24^ However, the roles of AMPK signalling‐mediated autophagy in OPG‐mediated inhibition of OC differentiation remain unclear.

## 2 MATERIALS AND METHODS

### 2.1 Reagents and antibodies

Dulbecco modified Eagle's medium (DMEM, 12800017, Gibco), α‐minimum essential medium (α‐MEM, 11900024, Gibco), foetal bovine serum (FBS, 10099‐141) and Lipofectamine™ 3000 Transfection Reagent (L3000015, Invitrogen) were obtained from Thermo Fisher Scientific. BAF (196000), TRAP staining kit (387A), DAPI staining solution (D9542), 2′,7′‐Dichlorofluorescin diacetate (D6883), 3‐methyladenine (M9281, 3‐MA) and LC3B antibody (L7543) were obtained from Sigma‐Aldrich. OPG (459‐MO), RANKL (315‐11) and M‐CSF (315‐02) were obtained from R＆D systems. EGFP‐pmCherry‐LC3 plasmid was obtained from HedgehogBio Science and Technology Ltd. Primary antibodies for c‐Fos (2250), Beclin1 (3495), p62 (8025), Atg5 (12994), Atg7 (8558), Atg12 (4180), phospho (p)‐mTOR (Ser2448, 5536), mTOR (2983), Raptor (2280), GβL (3274), p‐AMPKα (Thr172, 2535), AMPKα (2532), TSC2 (4308), Rheb (13879), p‐p70S6K (Thr421/Ser424, 9204), p70S6K (9202), β‐actin (4970L) and β‐actin, and (HRP)‐conjugated IgG were obtained from Cell Signaling Technology. Mouse monoclonal anti‐NFATc1 (sc‐7294) and CTSK (sc‐7294) antibodies, control siRNA (sc‐37007) and AMPKα1/2 siRNA (sc‐45313) were obtained from Santa Cruz Biotechnology. Com C (P5499), AICAR (Acadesine/AICA riboside, ab120358) and Phalloidin‐iFluor 555 Reagent‐CytoPainter (ab176756) were obtained from Abcam. BCA protein assay kit (P0009) and Alexa Fluor 488‐labelled goat anti‐rabbit (A0423)/mouse IgG (A0428) were obtained from Beyotime. All other reagents were available in our laboratory.

### 2.2 Cell culture

Bone marrow monocytes/macrophages (BMMs) were separated by flushing bone marrow cells from 4‐5‐week‐old male BALB/c mouse femurs and tibia as previously described.^24^ Animal experiment protocols were approved by the Animal Care and Use Committee of Yangzhou University (Approval ID: SYXK [Su] 2007‐0005). The BMMs were cultured with α‐MEM supplemented with 10% FBS and maintained in a humidified incubator at 37°C, in 5% CO_2_ for 12 hours. Adherent cells were removed, and non‐adherent cells were cultured with M‐CSF (30 ng/mL) and RANKL (60 ng/mL) to obtain OC (as primary OC) for subsequent experiments. RAW264.7 (murine monocyte/macrophage cell line) cells were purchased from the American Type Culture Collection and grown in DMEM supplemented with 10% FBS. OC‐like cells (OCLs) were generated and differentiated in culture with α‐MEM supplemented with 10% FBS with the addition of M‐CSF (30 ng/mL) and RANKL (60 ng/mL). All of medium was replaced every 2 days.

### 2.3 Osteoclasts differentiation staining

The cells were fixed in 4% paraformaldehyde for 10 minutes at room temperature and stained using a TRAP staining kit. TRAP‐positive multinucleated cells containing were considered OCs and were counted by using a normal inverted microscope (Leica).

### 2.4 Transmission electron microscopy (TEM) observation of autophagosomes

Autophagy is a process in which cytoplasmic proteins or damaged organelles are packaged in double‐membraned vesicles that fuse with lysosomes to form autolysosomes to enable organelle renewal and cell metabolism. Sample preparation was performed as previously described.^24^ Autophagosomes/autolysosomes were observed by TEM following culture with different treatments.

### 2.5 Light chain protein 3 plasmid and small interfering RNA (siRNA) transfection

Osteoclasts were transfected with EGFP‐pmCherry‐LC3 plasmid (500 ng/well) for 12 hours and subjected to different treatments. The cells were fixed and treated with anti‐quenching agent in cell culture slides. LC3 puncta (EGFP‐LC3 represents autophagosomes and pmCherry‐LC3 represents autolysosomes) were identified using a TCS SP8 STED high‐resolution laser confocal microscope (Leica).

The siRNA targeting sequences (5′‐3′) are as follows: AMPKα1/2, sense: GCUAUCUUCUGGACUUCAA, antisense: UUGAAGUCCAGAAGAUAGC. As a control siRNA, we used a missense siRNA. Cells were transfected with siRNAs using Lipofectamine™ 3000 Transfection Reagent. Cell culture medium was then replaced with fresh α‐MEM medium incubated for 3 days. The medium was replaced every 2 days. At the end of incubation, various treatments were applied.

### 2.6 Flow cytometric analysis

The differentiated cells were stained with ROS fluorescent probe (2′,7′‐dichlorofluorescin diacetate) after different concentrations of OPG treatment for 3 hours. Cells were harvested, washed, fixed, permeabilized and then detected by LSRFortessa flow cytometer (BD Company). The data were analysed on a Flowjo cytometry system.

### 2.7 Western blotting

Cellular protein content was extracted using the BCA protein assay kit. Samples (20‐40 μg) were separated using SDS‐PAGE gels and transferred onto polyvinylidene difluoride (PVDF) membranes (ISEQ00010, Merck Millipore) by electrophoresis. PVDF membranes were blocked with 5% milk blocking buffer (in TBST solution) and then incubated with the primary antibodies overnight at 4°C. Subsequently, the PVDF membranes were incubated with second antibodies. PVDF membranes were scanned using Tanon 5200 electrochemiluminescence (ECL) detection system for quantitative analysis of protein expression.

### 2.8 Statistical analysis

Experimental data were analysed using analysis of variance (ANOVA); statistical significance of data was determined using one‐way ANOVA and SPSS 21.0 software (IBM SPSS). *P* < .05 and *P* < .01 were considered significantly different and highly significantly different results, respectively. All experiments were performed at least in triplicate.

## 3 RESULTS

### 3.1 AICAR alleviates OC differentiation and accumulation of LC3 puncta

**Figure 1:**
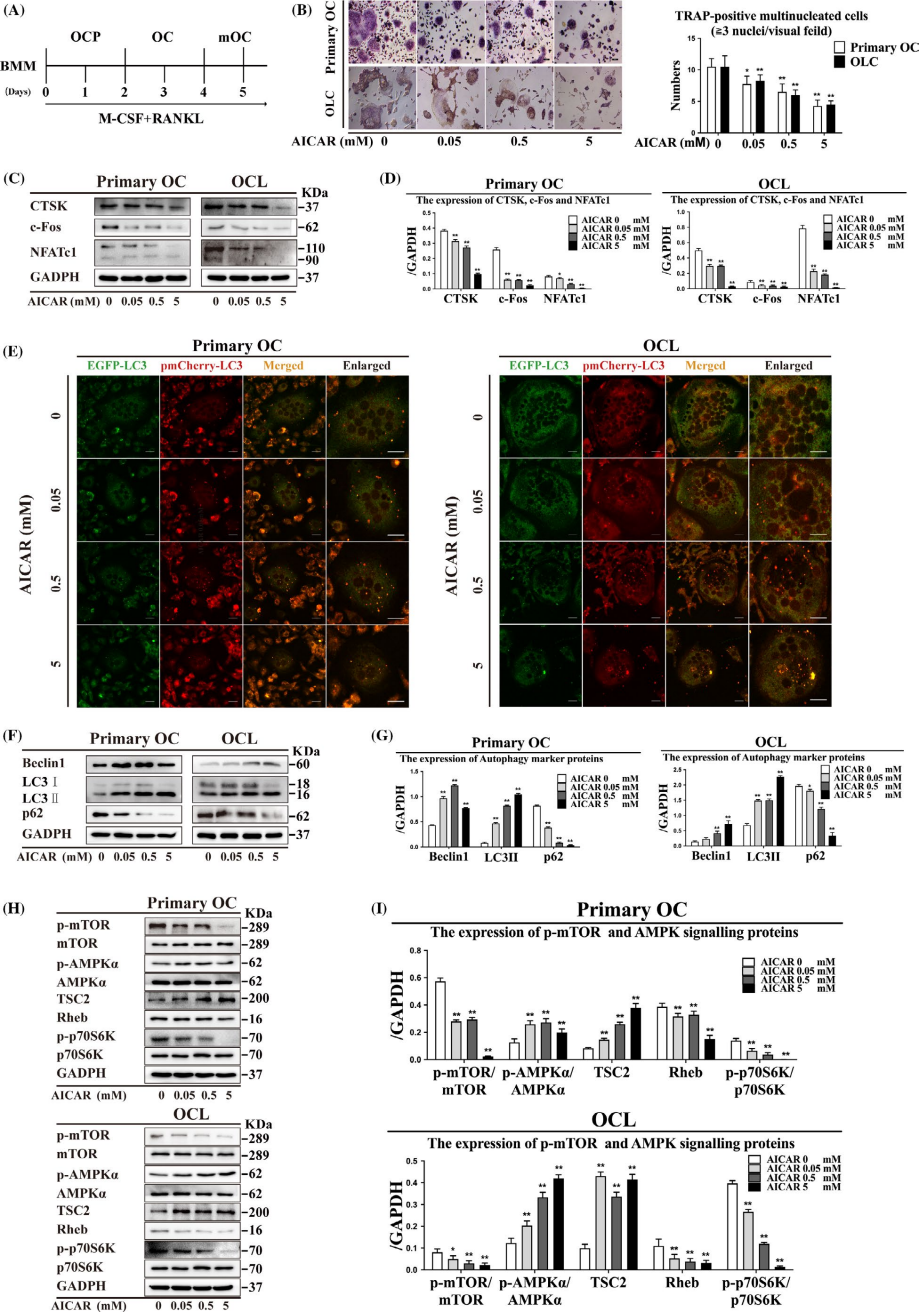
AICAR activates AMPK signalling by enhancing autophagy to reduce the number of TRAP‐positive multinucleated cells in primary OCs and OCLs. (A) OCP (derived from BMMs or RAW264.7 cells) were differentiated into OC and mature OC (mOC) models by supplementation with M‐CSF and RANKL. (B) TRAP‐positive multinucleated cells were observed following AICAR treatment for 3 h (n = 5). Magnification ×200. Scale bar = 20 μm. (C) OC differentiation marker protein expression was detected by Western blot after AICAR treatment for 3 h and (D) quantitatively analysed. (E) LC3 puncta were observed in differentiated OCs by laser confocal microscopy after AICAR treatment for 3 h. Magnification ×630. Scale bar = 20 μm. (F) Autophagy marker protein expression and (H) AMPK signalling proteins after AICAR treatment for 3 h were detected by Western blot and (G, I) quantitatively analysed. All data show means ± SD (n = 3). **P < .01 or *P < .05 vs. AICAR (0 mmol/L)

OCs formation gather from OCPs in bone reconstruction units, which then OCPs differentiate into multinucleated OCs by M‐CSF and RANKL induced (Figure 1A). AICAR (AMPK activator) is an analog of AMP and is capable of stimulating AMPK activity. TRAP enzyme has highly expressed in OC and is also as a flag for OC formation. Therefore, the test of TRAP enzyme is usually as a classic method of most related OC research. The number of TRAP‐positive multinucleated OCs was reduced after AICAR treatment (Figure 1B). The expression of CTSK, c‐Fos and NFATc1 was decreased by different concentration of AICAR (0.05, 0.5 and 5 mmol/L) treatment (Figure 1C, 1). LC3 puncta were accumulated following AICAR treatment (Figure 1E). These data indicated that AICAR treatment resulted in the decrease of OC differentiation and the increase of LC3 puncta.

### 3.2 AICAR activates autophagy and the AMPK signalling pathway during OC differentiation

We analysed the expression of autophagy marker proteins (Beclin1, LC3II and p62) after AICAR treatment in primary OCs and OCLs. Beclin1 and LC3II expression was upregulated, and p62 expression was decreased after AICAR treatment (Figure 1F, G). Moreover, we detected the expression of p‐mTOR/mTOR and AMPK signalling proteins in primary OCs and OCLs. P‐AMPKα/AMPKα and TSC2 expression was increased, and p‐mTOR/mTOR, Rheb and p‐p70S6K/p70S6K expression was decreased after AICAR treatment (Figure 1H, I). Our results indicated that autophagy and AMPK signalling were activated by AICAR treatment during OC differentiation.

### 3.3 Osteoprotegerin treatment causes decreased OC differentiation and accumulation of LC3 puncta

**Figure 2:**
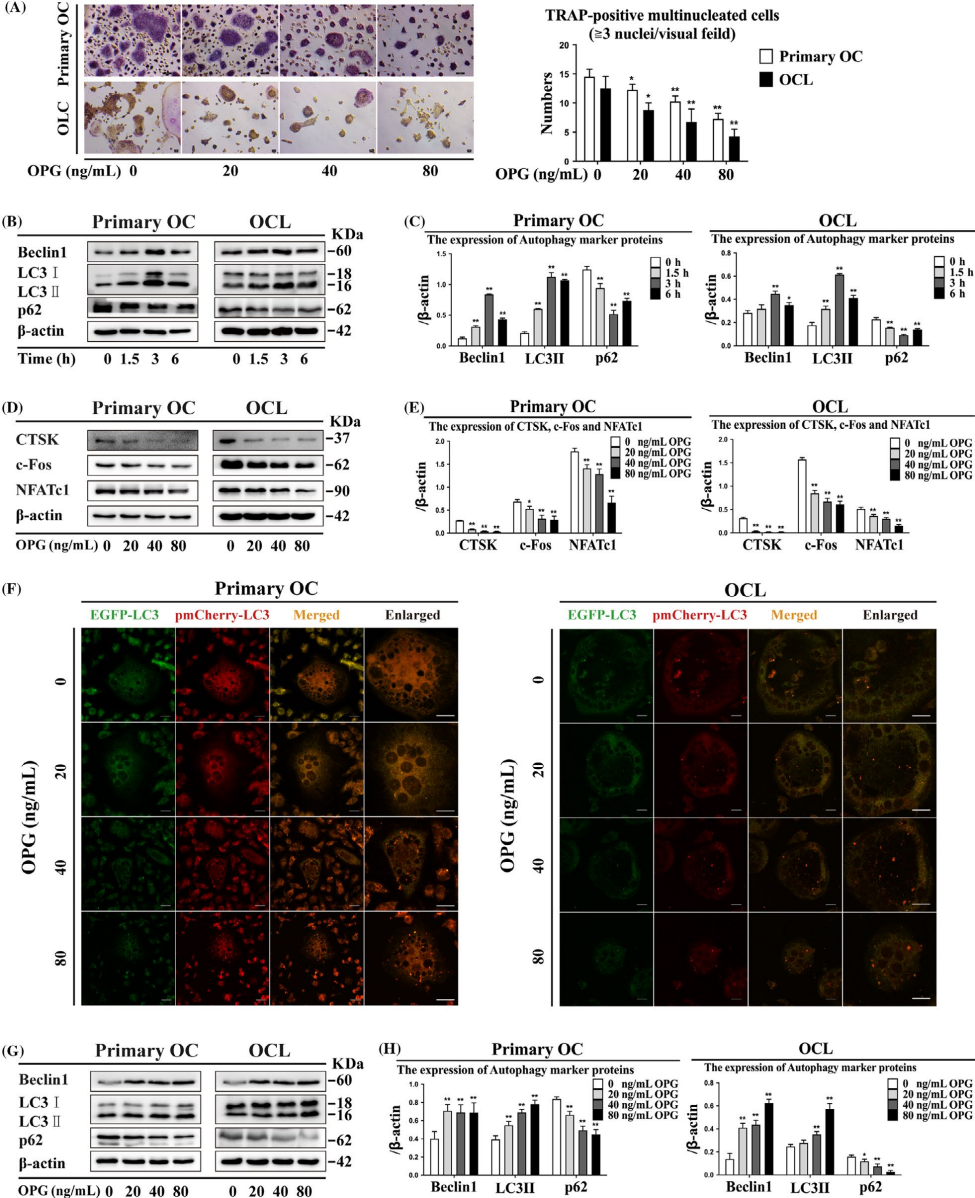
Osteoprotegerin enhanced autophagy during inhibition of OC differentiation in primary OCs and OCLs. (A) TRAP‐positive multinucleated cells were observed after OPG treatment for 3 h (n = 5). Magnification ×200. Scale bar = 20 μm. (B) The expression of autophagy marker protein and (D) OC differentiation marker protein was detected by Western blot at different treatment times (0, 1.5, 3 and 6 h) and OPG concentration and (C, E) quantitatively analysed. (F) LC3 puncta were observed by confocal microscopy during OPG‐mediated inhibition of OC differentiation. Magnification ×630. Scale bar = 20 μm. (G) Autophagy marker protein expression was detected by Western blot at different concentrations of OPG treatment for 3 h and (H) quantitatively analysed. All data show means ± SD (n = 3). **P < .01 or *P < .05 vs. OPG (0 ng/mL)

The number of TRAP‐positive multinucleated OCs decreased after OPG treatment in primary OCs and OCLs (Figure 2A). Beclin1 and LC3II expression was increased, and p62 expression was decreased after 40 ng/mL OPG treatment for 3 hours in primary OCs and OCLs (Figure 2B, 2). The expression of CTSK, c‐Fos and NFATc1 was decreased after OPG treatment in primary OCs and OCLs (Figure 2D, 2). LC3 puncta accumulated after OPG treatment in primary OCs and OCLs (Figure 2F). These data proved that OPG inhibits the number of TRAP‐positive multinucleated OCs, and the expression of CTSK, c‐Fos and NFATc1 was reduced by enhancing the expression of autophagy marker proteins and accumulation of LC3 puncta.

### 3.4 Osteoprotegerin treatment increases autophagy‐related proteins expression, number of autophagosomes and AMPK signalling molecules

**Figure 3:**
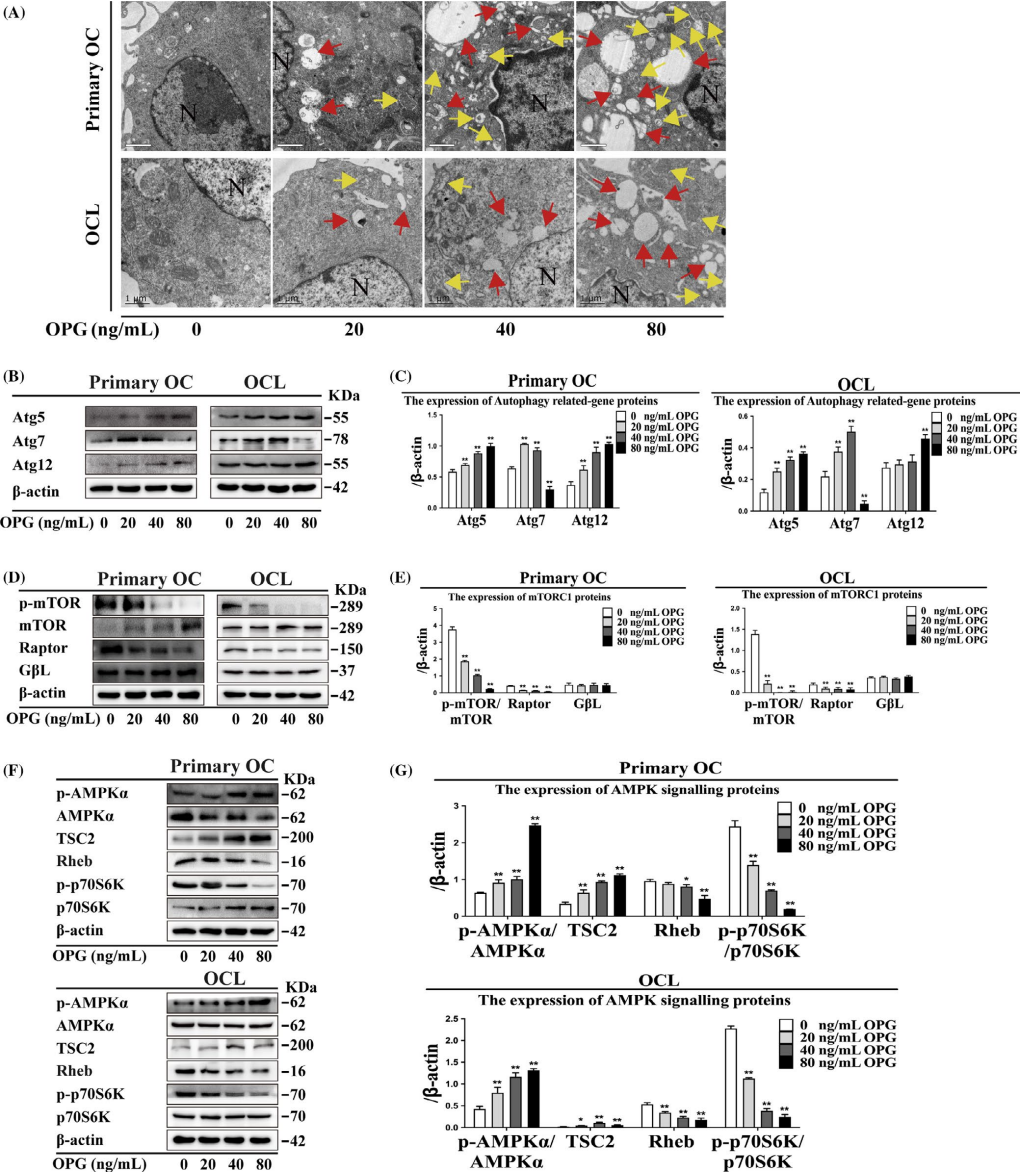
Autophagosome formation, suppression of mTORC1 and activation of AMPK signalling are involved in OPG‐mediated inhibition of OC differentiation in primary OCs and OCLs. (A) Autophagosome and autolysosome formation was observed by TEM during OPG‐mediated inhibition of OC differentiation. Magnification ×6600. Scale bar = 0.5 μm. N: nucleus; yellow arrows: autophagosomes; red arrows: autolysosomes. (B‐G) Expression of autophagy‐related genes; mTORC1 and AMPK signalling proteins were detected by Western blot and quantitatively analysed during OPG‐mediated inhibition of OC differentiation. All data show means ± SD (n = 3). **P < .01 or *P < .05 vs. OPG (0 ng/mL)

Beclin1 and LC3II expression was increased after OPG treatment in primary OCs and OCLs (Figure 2G, H). Autophagosome and autolysosome formation increased after different concentrations of OPG treatment in primary OCs and OCLs (Figure 3A). Atg5 and Atg12 expression increased after all OPG treatments, and Atg7 expression decreased after 80 ng/mL OPG treatment in primary OCs and OCLs (Figure 3B, 3). P‐AMPKα/AMPKα and TSC2 expression increased, and p‐mTOR/mTOR, Raptor, Rheb and p‐p70S6K/p70S6K expression decreased after OPG treatment in primary OCs and OCLs (Figure 3D‐G). These data indicated that the expression of autophagy‐related proteins and the number of autophagosomes increased, and AMPK signalling molecules were activated by OPG treatment.

### 3.5 3‐methyladenine reduces OC differentiation and enhances LC3 puncta

**Figure 4:**
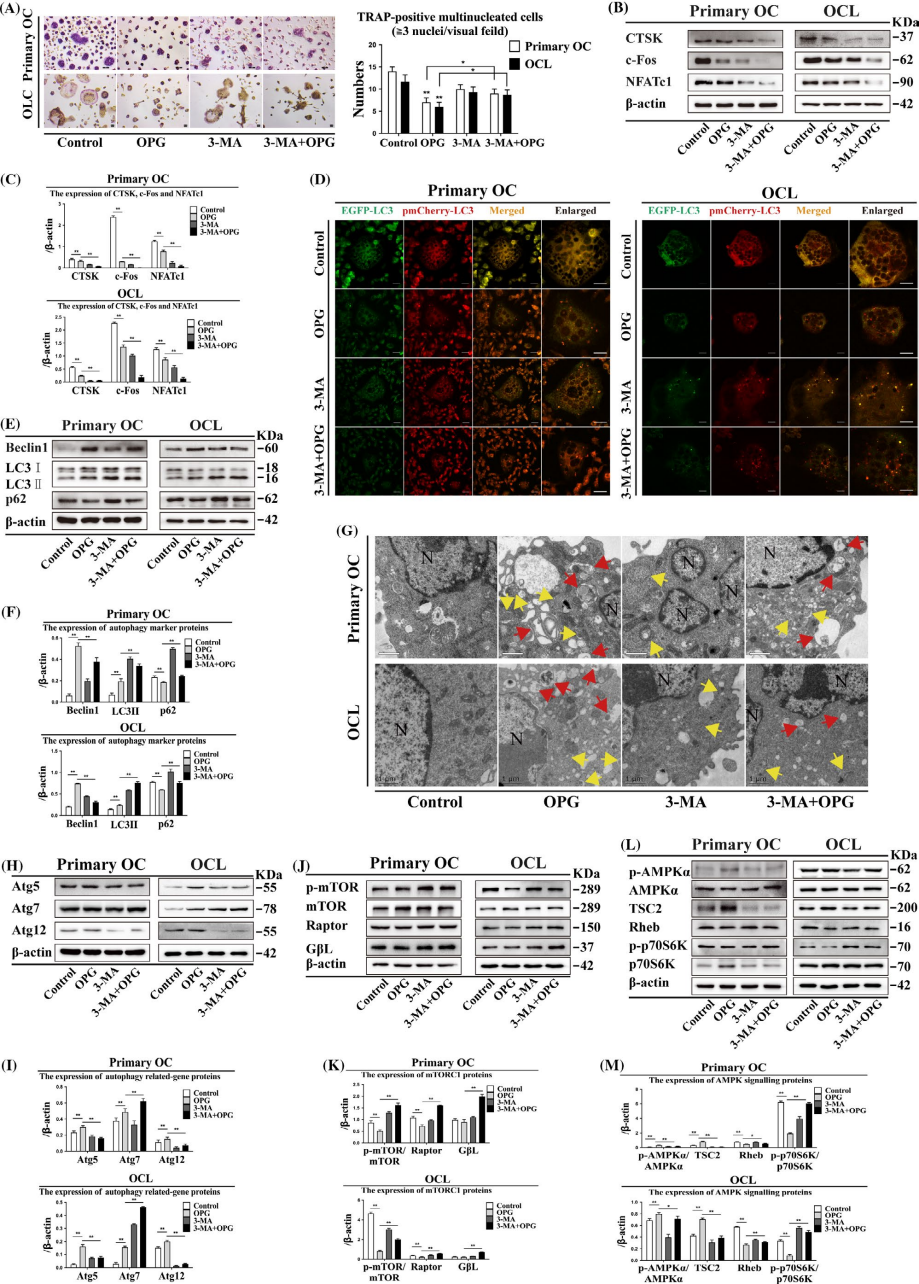
3‐methyladenine attenuates autophagy and AMPK signalling and activates mTORC1 during OPG‐mediated inhibition of OC differentiation in primary OCs and OCLs. (A) TRAP‐positive multinucleated cells were observed following OPG treatment with or without 3‐MA for 3 h (n = 5). Magnification ×200. Scale bar = 20 μm. (B) OC differentiation markers were detected by Western blot and (C) quantitatively analysed after OPG treatment with or without 3‐MA for 3 h. (D) LC3 puncta were observed by confocal microscopy during OPG treatment with or without 3‐MA for 3 h. Magnification ×630. Scale bar = 20 μm. (E) Expression of autophagy marker proteins, (H) autophagy‐related proteins, (J) mTORC1 and (L) AMPK signalling were detected by Western blot after OPG treatment with or without 3‐MA for 3 h and (F, I, K and M) quantitatively analysed. (G) Formation of autophagosomes and autolysosomes were observed by TEM during the OPG treatment with or without 3‐MA for 3 h. Scale bar = 1 μm. N: nucleus; yellow arrows: autophagosomes; red arrows: autolysosomes. All data show means ± SD (n = 3). **P < .01 or *P < .05

3‐methyladenine is a common inhibitor of autophagy, which inhibits PI3K activity and pre‐autophagosome formation. The number of TRAP‐positive multinucleated OCs decreased after 3‐MA treatment; however, the 3‐MA + OPG group showed more TRAP‐positive multinucleated OCs than did the OPG‐treated group (Figure 4A). CTSK, c‐Fos and NFATc1 expression decreased, and LC3 puncta accumulated after 3‐MA treatment in primary OCs and OCLs (Figure 4B, 4, 4). These data indicated that the inhibition of autophagy and accumulation of LC3 puncta by 3‐MA treatment caused reduced OC differentiation.

### 3.6 3‐methyladenine reduces autophagosome count, Atg5 and Atg12 expression and AMPK signalling

Light chain protein 3 specifically localizes to autophagosome membranes. Beclin1 expression decreased, and LC3II and p62 expression increased in the 3‐MA + OPG group compared with that in the OPG group in primary OCs and OCLs (Figure 4E, 4). However, the number of autophagosomes was lower after 3‐MA + OPG treatment than that after OPG treatment in primary OCs and OCLs (Figure 4G). Atg5 and Atg12 expression decreased, and Atg7 expression increased after 3‐MA + OPG treatment compared with that after OPG treatment (Figure 4H, I). mTORC1 (p‐mTOR/mTOR, Raptor and GβL) expression was higher after 3‐MA + OPG treatment than after OPG treatment in primary OCs and OCLs (Figure 4J, K). P‐AMPKα/AMPKα and TSC2 expression decreased, and Rheb and p‐p70S6K/p70S6K expression increased after 3‐MA + OPG treatment compared with that after OPG treatment (Figure 4L, M). These data demonstrated that 3‐MA treatment reduces the autophagosome count and weaken the expression of several proteins including Atg5, Atg12 and the proteins related to AMPK signalling pathway.

### 3.7 Bafilomycin attenuates OC differentiation, preserves bone resorption and enhances LC3 puncta formation

**Figure 5:**
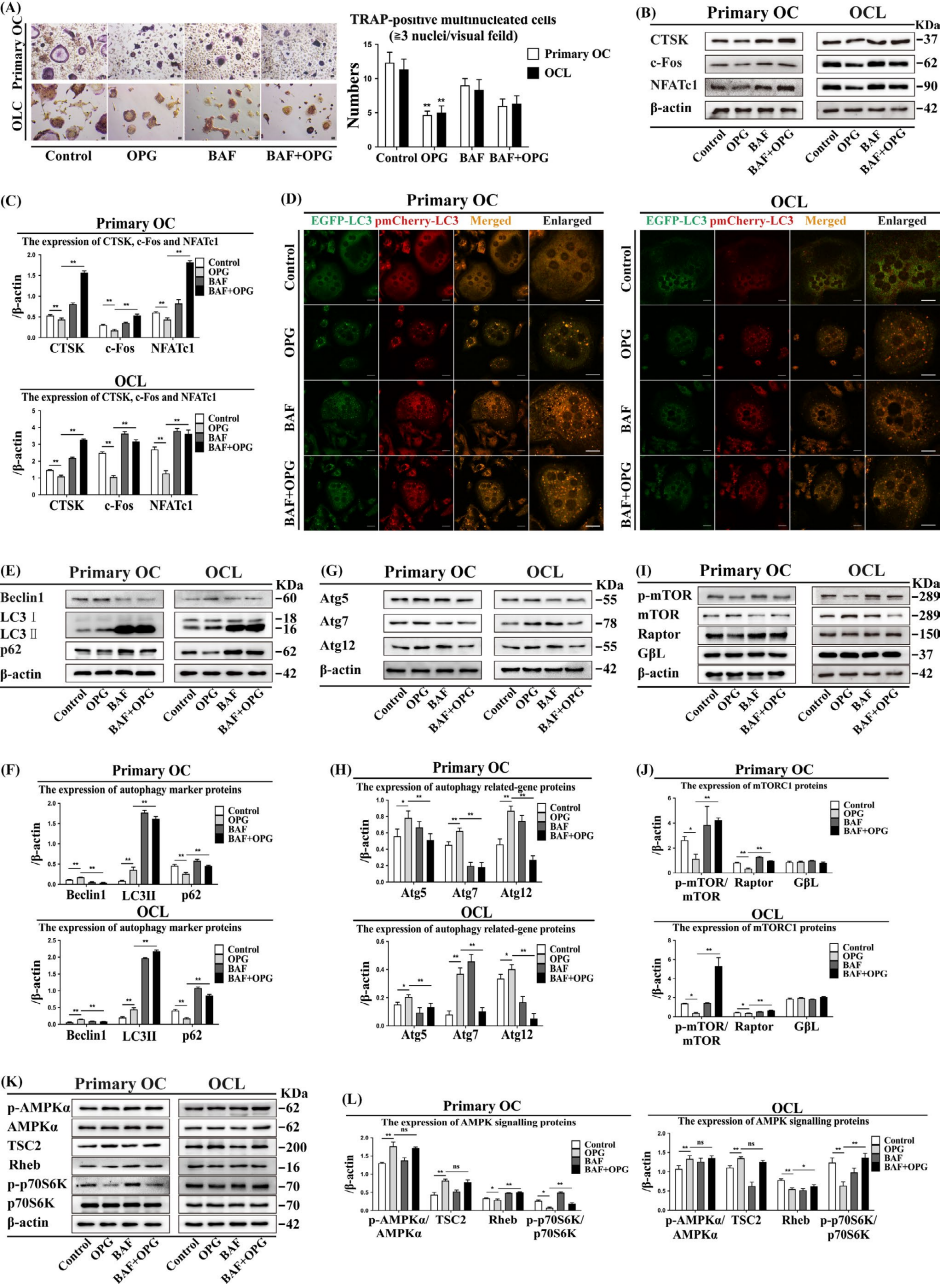
Bafilomycin attenuates autophagy by inducing mTORC1 activation and does not affect AMPK signalling. Suppression of OPG inhibits OC differentiation in primary OCs and OCLs. (A) TRAP‐positive multinucleated cells were observed after OPG treatment with or without BAF for 3 h (n = 5). Magnification ×200. Scale bar = 20 μm. (B) OC differentiation markers were detected by Western blot after OPG treatment with or without BAF for 3 h and (C) quantitatively analysed. (D) LC3 puncta were observed by confocal microscopy during the OPG treatment with or without BAF for 3 h. Magnification ×630. Scale bar = 20 μm. (E) Autophagy marker proteins, (G) autophagy‐related proteins, (I) mTORC1 and (K) AMPK signalling were detected by Western blot after OPG treatment with or without BAF for 3 h and (F, H, J and L) quantitatively analysed. All data show means ± SD (n = 3). **P < .01 or *P < .05

Bafilomycin is a potent‐specific V‐ATPase inhibitor that prevents the acidification of vesicles and inhibits cell fusion and multinucleation. The size and number of TRAP‐positive multinucleated OCs decreased after BAF treatment in primary OCs and OCLs (Figure 5A). CTSK and NFATc1 expression was higher, and c‐Fos expression lower after BAF + OPG treatment than after OPG treatment (Figure 5B, 5). BAF treatment also enhanced LC3 puncta formation in primary OCs and OCLs (Figure 5D). These data indicated that BAF treatment decreases the number of TRAP‐positive multinucleated OCs, but enhance the expression of CTSK, c‐Fos and NFATc1 and the formation of LC3 puncta.

### 3.8 Bafilomycin reduces Beclin1 and Atg expression, increases mTORC1, Rheb and p‐p70S6K/p70S6K expression, but does not affect of p‐AMPK/AMPK and TSC2

Bafilomycin inhibits late phase autophagy and can block the lysosome‐autophagosome fusion, leading to inactivation of acid degrading enzymes. Beclin1, Atg5, Atg7 and Atg12 expression was lower, and LC3II, p62, mTORC1, Rheb and p‐p70S6K/p70S6K expression was higher after BAF + OPG treatment than after OPG treatment in primary OCs and OCLs (Figure 5E‐L). However, there was no significant difference in p‐AMPK/AMPK and TSC2 expression between the various treatment groups (Figure 5K, L). These data indicated that BAF treatment could attenuate autophagy by reducing the expression of Beclin1 and Atg and increase mTORC1, Rheb and p‐p70S6K/p70S6K, but does not affect AMPK and TSC2.

### 3.9 Suppression or knockdown of AMPK attenuates OC differentiation and enhances LC3 puncta formation

**Figure 6:**
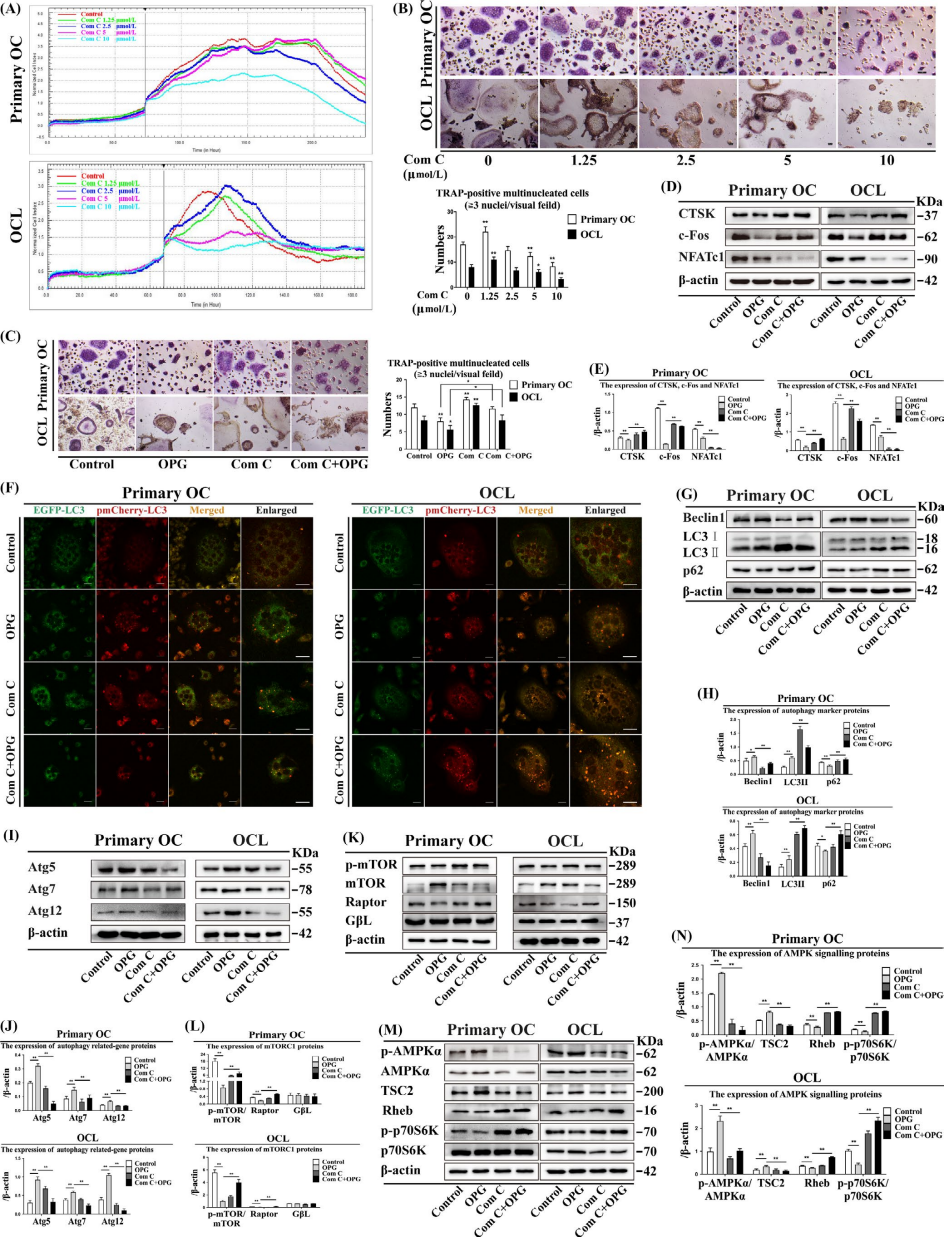
Com C decreases autophagy and AMPK signalling, and preserves bone resorption in primary OCs and OCLs by inducing mTORC1 activation during OPG‐mediated OC inhibition. (A) The kinetics of OC differentiation were determined at different concentrations of Com C. Cell index (CI) values were normalized to the effect of the treatment. Each treatment was repeated three times, and the average was plotted. Error bars denote SD (n = 5). (B, C) TRAP‐positive multinucleated cells were observed by different concentrations of Com C, and OPG treatment with or without Com C for 3 h respectively. Magnification ×200. Scale bar = 20 μm. (D) OC differentiation markers were detected by Western blot after OPG treatment with or without Com C for 3 h and (E) quantitatively analysed. (F) LC3 puncta were observed by confocal microscopy during the OPG treatment with or without Com C for 3 h. Magnification ×630. Scale bar = 20 μm. (G) Autophagy marker proteins, (I) autophagy‐related proteins, (K) mTORC1 and (M) AMPK signalling were detected by Western blot after OPG treatment with or without Com C for 3 h and (H, J, L and N) quantitatively analysed. All data show means ± SD (n = 3). **P < .01 or *P < .05

**Figure 7:**
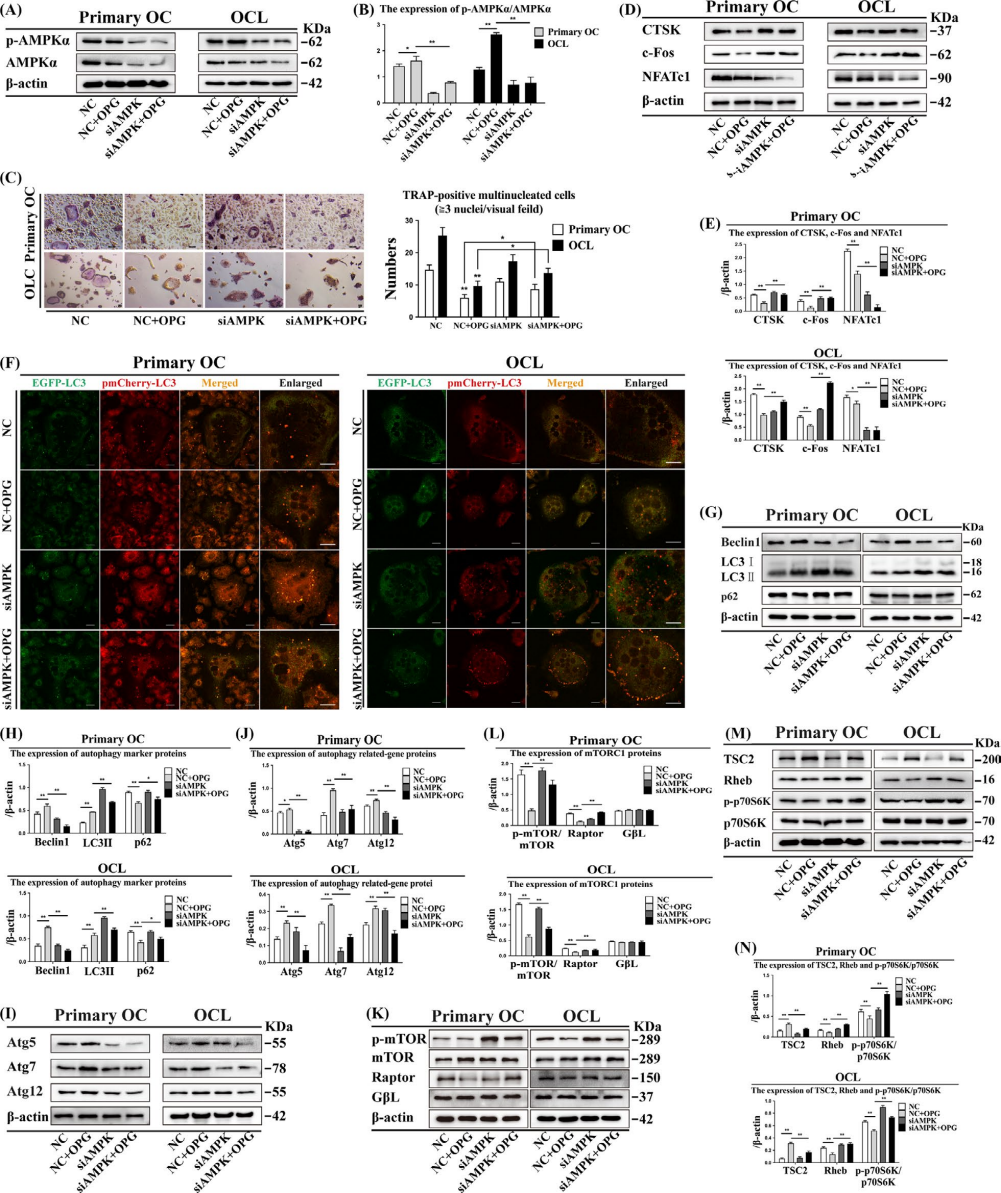
Knockdown of AMPK could attenuate autophagy and AMPK signalling by inducing mTORC1 activation and rescue bone resorption during OPG‐mediated inhibition of OC differentiation in primary OCs and OCLs. (A) Effect of AMPK knockdown on p‐AMPK/AMPK expression was detected by Western blot and (B) quantitatively analysed. (C) TRAP‐positive multinucleated cells were observed after OPG treatment with or without siAMPK for 3 h (n = 5). Magnification ×200. Scale bar = 20 μm. (D) OC differentiation markers were detected by Western blot after OPG treatment with or without siAMPK for 3 h and (E) quantitatively analysed. (F) LC3 puncta were observed by confocal microscopy following OPG treatment with/ without siAMPK for 3 h. Magnification ×630. Scale bar = 20 μm. (G) Autophagy marker proteins, (I) autophagy‐related proteins, (K) mTORC1 and (M) AMPK signalling were detected by Western blot after OPG treatment with or without siAMPK for 3 h and (H, J, L and N) quantitatively analysed. All data show means ± SD (n = 3). **P < .01 or *P < .05

We determined the kinetics of OC morphological changes after Com C treatment by using the real‐time xCelligence system (ACEA Biosciences, Inc). The cell index (CI) includes cell viability, number, degree of adhesion and morphology, and is a quantitative measure of cell status. CI was recorded in the presence of M‐CSF and RANKL with or without Com C. The CI values were not significantly different at different concentrations of Com C (1.25, 2.5 and 5 μmol/L) compared with the control group in primary OCs and OCLs, but were decreased after treatment with 10 μmol/L Com C (Figure 6A). The number of TRAP‐positive multinucleated OCs was decreased after treatment with 5 and 10 μmol/L Com C, but increased after treatment with 1.25 μmol/L Com C in primary OCs and OCLs (Figure 6B). OC size and number increased after Com C + OPG treatment in primary OCs and OCLs (Figure 6C). NFATc1 expression was lower, and CTSK and c‐Fos expression was higher after Com C + OPG treatment than after OPG treatment in primary OCs and OCLs (Figure 6D, 6). Com C treatment also increased LC3 puncta in primary OCs and OCLs (Figure 6F). AMPK knockdown had the same effects as AMPK suppression (Figure 7C‐F). These data showed that suppression or knockdown of AMPK attenuates OC differentiation and enhances the formation of LC3 puncta.

### 3.10 Suppression or knockdown of AMPK increases LC3 II, p62 and mTORC1 expression and inhibits AMPK signalling

Com C is an inhibitor of the key intracellular energy sensor AMPK and can induce autophagy.^37^ Expression of autophagy markers (LC3II and p62), mTORC1 (p‐mTOR/mTOR and Raptor), Rheb and p‐p70S6K/p70S6K was higher after Com C + OPG treatment than after OPG treatment in primary OCs and OCLs, while GβL expression was not significantly different (Figure 6G, H and Figure 6K‐N). In contrast, the expression of Beclin1, Atg5, Atg7, Atg12, p‐AMPKα/AMPKα and TSC2 was lower after Com C + OPG treatment than after OPG treatment in primary OCs and OCLs (Figure 6G‐J and Figure 6M‐N). Knockdown of AMPK had the same effects as AMPK suppression (Figure 7A, 7B and 7G‐N). These data suggested that suppression or knockdown of AMPK increases the expression of LC3 II, p62 and mTORC1, which are as the symbol of autophagy, but inhibits AMPK signalling.

## 4 DISCUSSION

AMPK is a metabolic energy sensor that plays a pivotal role in maintaining cellular energy homeostasis. Activation of AMPK induces phosphorylation of downstream signalling targets and inhibits OC differentiation and bone resorption.^32^, ^38^, ^39^ OC differentiation and bone resorption require a series of regulatory molecules and hydrolytic enzymes to establish dynamic homeostasis with OBs.^1^, ^40^ In this study, we used AICAR to activate AMPK and showed that OC differentiation was reduced by increased p‐AMPKα/AMPKα expression and phosphorylation of its downstream signalling proteins. Thus, AMPK is involved in OC differentiation via regulation of autophagy and its downstream signalling pathway.

Osteoprotegerin is a secreted glycoprotein that improves bone mass by inhibiting OC differentiation in vitro and in vivo.^6^, ^41^, ^42^ Liu et al^43^ reported enhanced root resorption due to increased OC activation and reduced cementum mineralization in OPG‐knockout mice in vitro and in vivo. OPG is a decoy receptor for RANKL and is a key physiological regulator in OC differentiation and function; RANKL is also produced by OBs in a membrane‐ligand form.^44^ Li et al^45^ demonstrated that autophagy is essential to the regulation of bone homeostasis in OB‐specific Atg7‐knockout mice and that defective autophagy in OBs led to reduced bone mass, increased OC number and triggered endoplasmic reticulum stress in OBs. Activation of autophagy promotes osteoclastogenesis and stimulates OC‐mediated bone resorption during hypoxia or RANKL‐induced OC differentiation, which involves the bridge protein p62. P62 knockdown inhibits autophagy activation in RANKL‐induced OC.^21^, ^46^ Our results indicated that OPG inhibits OC differentiation by enhancing autophagy and phosphorylation of AMPK downstream signalling proteins.

Previous study shown that RANKL simulation of BMMs transiently increased the level of intercellular ROS through a signalling cascade involving TRAF‐6/Rac1/NAPDH pathway.^7^ OC differentiation requires involvement of ROS to perform bone resorption by activation of MAPKs signalling.^7^, ^47^ In fact, overproduction of ROS causes oxidative stress that is a cellular damage, which results from lipid oxidation, membranes structural alteration and nucleic acids proteins oxidation, even become systemic diseases.^48^ ROS are a physiological activator for AMPK, and in turn, activation of AMPK limits mitochondria ROS production by peroxisome proliferator‐activated receptor gamma coactivator 1α (PGC1α)‐dependent antioxidant response. Interesting, AMPK‐PGC1α‐dependent control of ROS regulates hypoxia‐inducible factor 1α (HIF1α) stabilization.^49^ In addition, AMPK activity results in redox changes that is not due to directly action on AMPK itself, but is a secondary consequence of redox effects on other process (such as mitochondria ATP production).^50^ The present results demonstrated that the level of OC intercellular ROS has no changes significantly during OPG‐mediated inhibition of osteoclast differentiation (see the Figure S1).

Early autophagy involves many signalling molecules to activate downstream signalling pathways. 3‐MA, an autophagy inhibitor, and suppresses cytoplasmic LC3 I transfer into the autophagosome membrane protein LC3II to block the early initial steps of autophagy.^51^ RANKL‐induced OC differentiation significantly promotes expression of autophagy‐related markers including p62. However, during OC differentiation, p62 was significantly downregulated initially (probably owing to autophagy activation) and then gradually increased. In addition, p62 knockdown attenuated the expression of RANKL‐induced autophagy‐related gene and osteoclastogenesis‐related gene, TRAP‐positive multinuclear cell count and accumulation of LC3. 3‐MA significantly rescued p62 degradation during the initial stages of RANKL‐induced OC differentiation.^21^ In our study, 3‐MA treatment increased p62 expression during OPG‐mediated inhibition of OC differentiation and the TRAP‐positive multinuclear cell count and attenuated AMPK signalling pathway‐mediated autophagy.

Bafilomycin, a late‐stage lysosome inhibitor, could prevent lysosome‐autophagosome fusion, causing autophagic flux disruption.^52^ BAF binds to the Vo subunits of V‐ATPase to inhibit bone resorption activity, which results in OC apoptosis and the impairment of OC endocytosis.^53^ BAF suppresses the expression of NF‐κB and NFAT during RANKL‐induced OC differentiation, resulting in smaller and fewer OC nuclei, due to increased p62 expression.^30^ In our study, BAF treatment reduced the number of OCs produced by M‐CSF and RANKL treatment, and increased CTSK and NFATc1 expression, which is inconsistent with the results reported by Cai et al^54^ CTSK is a lysosomal cysteine protease capable of cleaving the helical and telopeptide regions of type I collagen during OC differentiation.^55^ Inactivation of NFATc1 by cyclosporin‐A treatment attenuated the expression of Atp6v0d2 and DC‐STAMP and the subsequent OC fusion process, and NFATc1 activity restored by overexpression of Atp6v0d2 and DC‐STAMP rescued cell‐cell fusion and OC differentiation.^56^ In this study, BAF treatment, suppressed V‐ATPase activation, did not rescue OC differentiation and increased NFATc1 expression, possibly owing to BAF‐mediated inhibition of V‐ATPase transport to the bone resorption lacunae, impeding CTSK degradation.

Com C, an AMPK inhibitor, was used to determine the relationship between the AMPK/mTOR/p70S6K signalling pathway and autophagy during OPG‐mediated inhibition of OC differentiation and bone resorption.^54^ AMPK, positively regulates autophagy, is activated during OPG‐mediated inhibition of OC differentiation, by suppression of mTOR and phosphorylation of the downstream target p70S6K. Active AMPK causes phosphorylation and activation of the TSC1/2 complex, which inhibits mTOR activity through Rheb.^57^ Com C prevented inhibition of p70S6K phosphorylation by mTOR. We used siRNA to knockdown AMPK, thereby causing raptor inactivation and subsequent upregulation of Rheb and phosphorylation of mTOR and p70S6K. NFATc1 and CTSK expression was decreased after Com C treatment and AMPK knockdown. These results are contrary to those of Lee et al^33^

**Figure 8:**
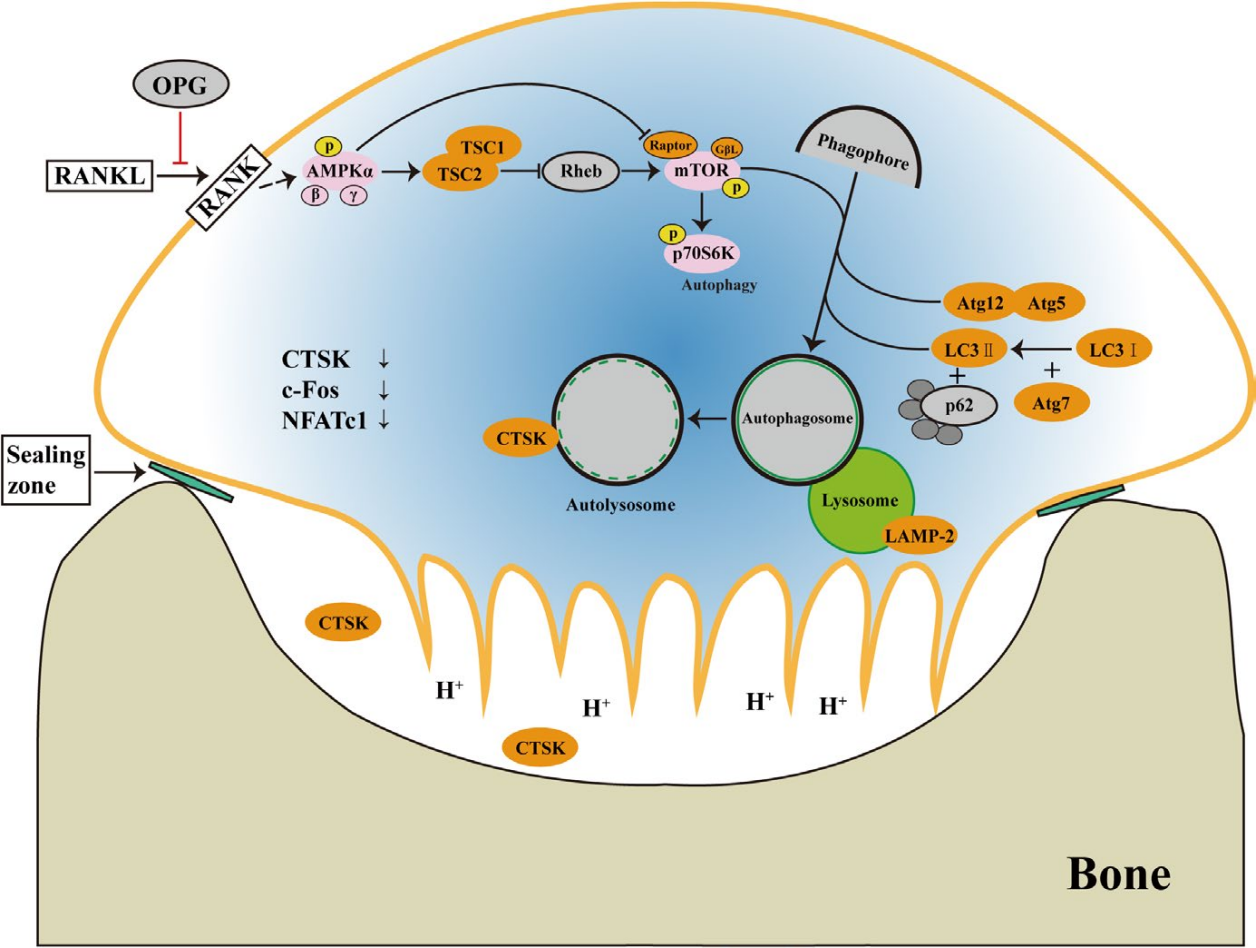
Schematic representation of OPG‐mediated inhibition of OC differentiation and bone resorption by induction of AMPK signalling‐mediated autophagy. OPG competitively combines with RANKL, causing the inhibition of OC differentiation, which is a key regulator of bone metabolism. Lysosomes, which secrete matrix proteases (such as CTSK) and regulate the function of OC by the control of pH, also play a role in this process

Additionally, we produced a schematic mode of OPG‐mediated inhibition of OC differentiation by the induced AMPK signalling pathway and autophagy. The AMPK signalling pathway is involved in OPG‐mediated inhibition of OC differentiation by inducing autophagy. Furthermore, lysosomes are pivotal organelles in OC differentiation and secrete matrix proteases (such as CTSK) to regulate the function of OC (Figure 8).

In conclusion, we demonstrate that the AMPK signalling pathway prominently regulates the OPG‐mediated inhibition of OC differentiation, by inducing autophagy. Our results provide a basis for future bone‐related studies on the AMPK signalling pathway.

## CONFLICT OF INTEREST

All authors declare that they have no conflict of interest.

## AUTHOR CONTRIBUTIONS

ZPL, JHG, JCB and XST conceived and designed the study. XST, Y.C, YGM and C.Z performed the experiments. JCB, HYZ and JHG contributed materials and reagents. XST, D.W and RLS analysed the data. XST and ZPL wrote the manuscript.

## Data Availability

The data that support the findings of this study are available from the corresponding author upon reasonable request.
