# Supplementary figures and images for: Suppression of AMP‐activated protein kinase reverses osteoprotegerin‐induced inhibition of osteoclast differentiation by reducing autophagy
Source: Cell Prolif. 2019 Nov 7;53(1):e12714. doi: 10.1111/cpr.12714 (PMC6985670; doi:10.1111/cpr.12714)

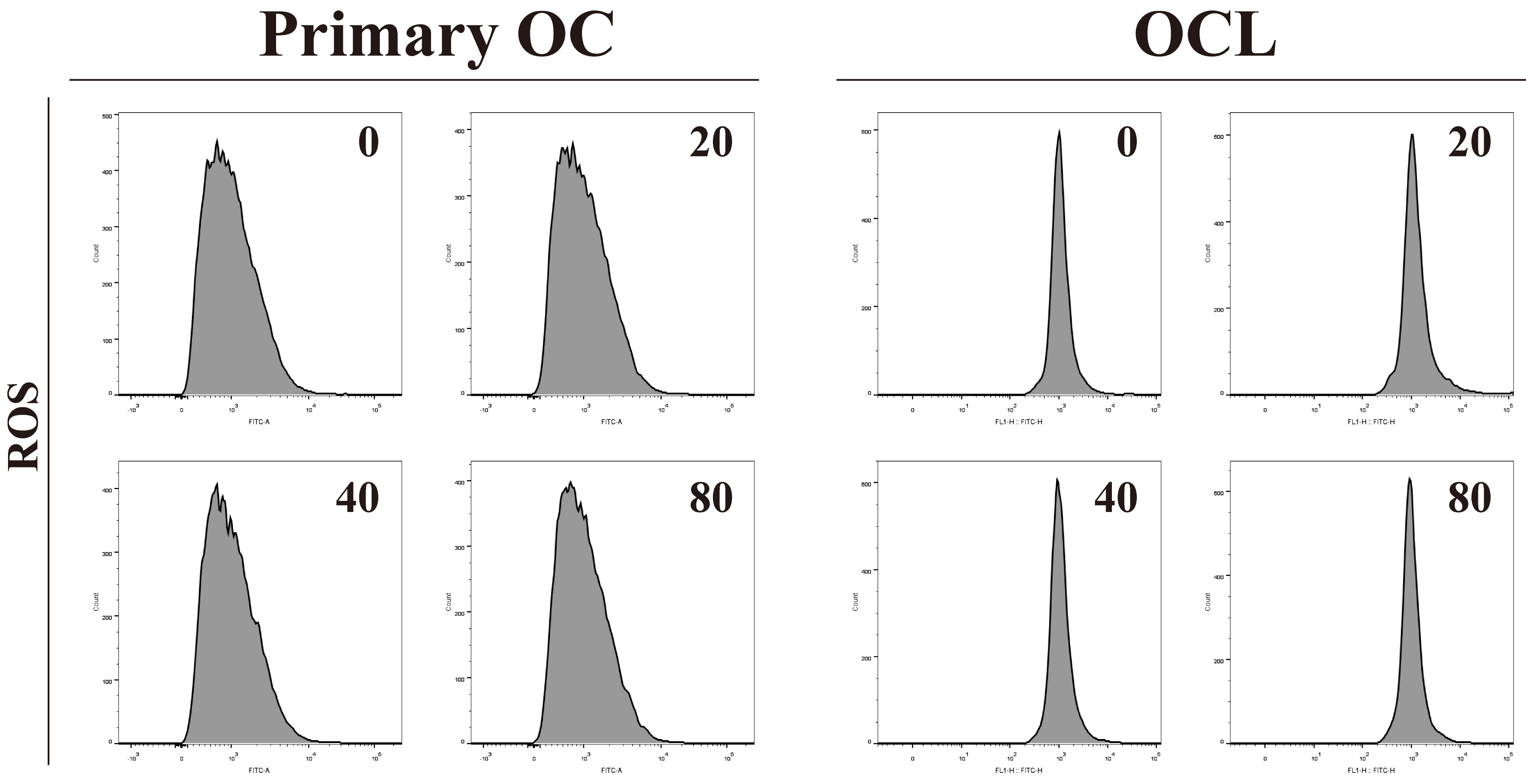

Supplement: Supplementary file 1 [file CPR-53-e12714-s001.tif]
